# Summary of presentations at the NIH/NIAID New Humanized Rodent Models 2007 Workshop

**DOI:** 10.1186/1742-6405-5-3

**Published:** 2008-01-31

**Authors:** Harris Goldstein

**Affiliations:** 1 Departments of Pediatrics and Microbiology & Immunology, Albert Einstein College of Medicine, Bronx, New York, 10461, USA

## Abstract

It has long been recognized that a small animal model susceptible to HIV-1 infection with a functional immune system would be extremely useful in the study of HIV/AIDS pathogenesis and for the evaluation of vaccine and therapeutic strategies to combat this disease. By early 2007, a number of reports on various rodent models capable of being infected by and responding to HIV including some with a humanized immune system were published. The New Humanized Rodent Model Workshop, organized by the Division of AIDS (DAIDS), National Institute Allergy and Infection Diseases (NIAID), NIH, was held on September 24, 2007 at Bethesda for the purpose of bringing together key model developers and potential users. This report provides a synopsis of the presentations that discusses the current status of development and use of rodent models to evaluate the pathogenesis of HIV infection and to assess the efficacy of vaccine and therapeutic strategies including microbicides to prevent and/or treat HIVinfection.

## Introduction

Investigation of many aspects of the in vivo behavior of HIV as well as testing of the in vivo efficacy of novel anti-HIV therapies and vaccines has been hampered by the restriction of HIV infection to humans and primates [1]. Mice cannot be infected with HIV-1, because sequence differences in mouse homologues of the human proteins required for HIV replication prevent their interaction with essential HIV proteins critical for HIV replication such as Env, Tat [2,3] and Rev [3,4], as well as prevent and potentially limit efficient assembly and budding of virus from the cell membrane. These genetic differences result in blocks at several stages of HIV replication that prevents cellular infection and efficient production of HIV-1 by mouse cells.

It has long been recognized that a small animal model with a reconstituted human immune system would be extremely useful in the study of HIV/AIDS pathogenesis and for the evaluation of vaccine and therapeutic strategies to combat this disease. By early 2007, a number of reports on rodent models with a humanized immune system capable of being infected by and responding to HIV were published. The New Humanized Rodent Model Workshop, organized by Janet Young, Paul Black, Tony Conley, Jim Turpin, Fulvia Veronese and Opendra Sharma from DAIDS, NIAID, NIH, was held on September 24, 2007 at Bethesda for the purpose of bringing together key model developers and potential users. The meeting included a discussion by a panel about the current status of the models, future plans, as well as potential use of the models for addressing critical issues in basic immune response studies, pathogenesis, therapeutics, vaccines and microbicides development. Speakers were asked to address the following questions:

### Model advantages

What unique advantages does your model offer over the other recently reported humanized mouse and rat models versus SCID-hu and HuPBL-SCID, and existing non-human primate models?

### Possible studies

*What types of studies does your model permit that were not possible previously? Can you expand on categories of studies, for example. therapeutics, vaccines, PrEP, PEP, pathogenesis, immunology studies, prevention, and microbicides*.

### Limitations

What are the limitations of your model? Be honest!

### Cohort size

*What size cohorts of mice can you routinely make? How many reconstituted mice can you make per week/month? Are these available to other investigators? If not, what are the limitations? How consistent and reproducible are reconstitution and infection in your system? Please provide percentages of success for infection and numbers of mice that can be generated (week or month) based on your experience*.

### Model availability

*How widely is your system, available especially the strain of mice used? Who supplies your mice? Is your mouse strain commercially available? If it is commercially available and you do not use it please explain why not*.

### Stem cells and fetal tissue

Please provide the following details about the stem cells/fetal tissue used for the model: source and availability; amount needed for your model; can they be pooled from multiple donors; and must the cells/tissue be fresh or can they be frozen?

### Model development

*Please provide the details of the model development. We are particularly interested in parameters such as titer of inoculating virus, characteristics of the virus(s) used (strain, source), use of cell-free and/or cell-associated virus, if a laboratory isolate or a clinical isolate is used, and what clades, routes of inoculation, and efficiency of infection (methods and ranges for the endpoint) have been used and measured*.

### Human cell distribution

What are the identity (including subsets R5/X4 expression), functionality, and tissue distribution of subtypes of human immune cells in blood and at mucosal sites? Please include information on the female reproductive tract, rectum, lung, and GALT in these models and variations from animal to animal. How do these parameters compare to similar human sites? If your model does not have a human thymic epithelium, how do immature T cells get educated (positive and negative selection)?

### Current scientific studies

Provide a brief overview of some of the scientific studies that have been possible with your model so far. Unpublished data is encouraged!

### HLA restriction and antibody responses

Are there human HLA-restricted CD4 or CD8 responses? Are there antigen-specific human antibody responses and how do antibody titers compare with responses in humans?

Two broad approaches have been used to circumvent the replication blocks in rodents to generate small animal models for studying HIV infection. One broad approach used transgenic techniques to generate mice or rats capable of supporting HIV replication either by introducing transgenes encoding the human proteins critical for HIV replication or an HIV provirus into the genome of rodents. At the workshop, Drs. Littman, Keppler and Goldstein discussed these approaches. A second increasingly adopted approach utilizes chimeric human/mouse models to circumvent the inability of mouse cells to support HIV replication by transplanting human hematopoietic cells and/or human thymic tissues and/or fetal liver into immunodeficient mice. A model initially described by the McCune group designated either the SCID-hu mouse [5] or the thy-liv SCID mouse was constructed by surgically implanting fragments of human fetal thymus and liver under the kidney capsule of a SCID mouse. Two to three months after implantation, a thymus-like conjoint human organ grows which supports long-term multi-lineage hematopoiesis that leads to maturation of human thymocytes [6]. If sufficient thymic tissue is implanted, human T-cells are found in the peripheral blood for over a year, but no mature B cells are generated [7,8]. Injection of HIV-1 into the implant results in the killing of human thymocytes and the severe depletion of human CD4+ cells in the implant within a few weeks as well as plasma viremia. A limitation of this model is that no humoral or cellular responses to the HIV infection, including primary immune responses, occur in these chimeric mice [6,9]. This model is also limited by its construction using implanted tissues that are of fetal origin whose response to infection may not necessarily reflect the course of HV infection in patients where HIV predominantly infects lymph nodes and the gut associated lymphoid tissues. In a presentation at the workshop Dr. Stoddart discussed the current status and uses of this model. The chimeric human/mouse approach has been expanded by the recent description of new models that take advantage of novel mouse strains, Rag2^-/-^γ_c_^-/- ^mice and NOD/SCID/IL2Rγ^null ^mice, that are more immunodeficient than SCID mice and support engraftment and maturation of human hematopoietic stem cells into human T cells, B cells, monocytes and dendritic cells after injection with human CD34+ hematopoietic stem cells [10-16]. Drs. Akkina, Luban, Su, and Speck discussed their experiences with models that use Rag2^-/-^γ_c_^-/- ^mice and Dr. Shultz has included his experience with a model that uses NOD/SCID/IL2Rγ^null ^mice. Another chimeric human/mouse model was discussed by Dr. Martinez that combines human thymic implantation and transplantation with human HSC by implanting NOD/SCID mice with fetal liver and thymus and then transplanting them with syngeneic human CD34+ hematopoietic stem cells [17]. A synopsis of these presentations and Tables summarizing the techniques used to construct these models, the major features of the different models and the specific experiences of individual investigators using these models for HIV-related studies are presented below.

## Transgenic rodent models

Dr. Littman (Howard Hughes Research Institute, New York University Medical Center) has been working on utilizing transgenic approaches to overcome the replication barriers that prevent HIV infection of murine T cells (Table 1). These include the inability of HIV to enter mouse cells, the subsequent inefficient support of Tat-mediated trans-activation, the aberrant processing of HIV-Gag protein and the defective virion budding in mouse cells. To overcome the entry block, Dr. Littman used transcriptional regulatory sequences from mouse and human CD4 genes to construct transgenic mice expressing human CD4 and CCR5 in mouse CD4+ T cells, myeloid cells, dendritic cells and microglia. Using a Vpr-β-lactamase assay, his group demonstrated that HIV could efficiently enter into activated CD4+ T cells from these hCD4/CCR5 transgenic mice. After entry, he demonstrated that RT was functional in primary mouse T cells as indicated by the efficient generation of nuclear 2-LTR circles. The block due to inefficient Tat-mediated trans-activation is related to structural differences between mouse and human cyclin T1 (hCyclin T1), a protein which is required for Tat function and efficient HIV replication. A single amino acid difference at position 261 in mouse cyclin T1 (mCyclin T1) compared to hCyclin T1 prevents mCyclin T1 from binding of HIV Tat. The Littman group constructed mice transgenic for hCyclin T1 under the control of the CD4 promoter and crossed them with hCD4/CCR5 mice. Although expression of hCyclin T1 was associated with a several-fold increase in the production of HIV by mouse cells also expressing CD4 and CCR5, HIV RNA levels in the infected hCycT1 mouse T cells were still 10-fold lower than human cells. Efficient infection of mouse T cells required continued activation of the TCR with anti-CD3/CD28, particularly for the 12–20 hour period after infection. HIV production by mouse cells is also limited by a processing defect in the conversion of the gag p55 precursor to p24, leading to decreased production of p24 antigen which is required for construction of the viral capsids. Furthermore, the HIV produced by the mouse cells was less infectious than HIV produced by human cells. Electron microscopy demonstrated the abnormal budding of HIV in infected mouse T cells into the nuclear envelope and not the cell membrane. Murine Apobec3 cannot interact with HIV Vif, and hence can also inhibit HIV production by mouse cells, but this has not yet been fully assessed in murine T cells. This transgenic mouse model therefore does not support sufficient levels of HIV replication for pathogenesis, drug or vaccine studies.

**Table 1:** Transgenic Rodent Models

	**Dr. Goldstein**	**Dr. Keppler**	**Dr. Littman**
**Characteristics of Humanized Rodent Models**			
Strain	Full-length LTR-regulated HIV provirus and CD-promoter regulated human cyclin T1 expressed as transgenes in mice	Human CD4, CCR5 and cycin T 1 expressed as transgenes in Sprague-Dawley rats	Human CD4, CCR5 and cycin T 1 expressed as transgenes in mice
# mice/donor	NA	NA	NA
Source of human cells	NA	NA	NA
Method of isolation	NA	NA	NA
Pre-transplant treatment-mice	NA	NA	NA
Pre-transplant treatment-cells	NA	NA	NA
Time frame from construction to experimental use	immediately	Immediately	immediately
Location of human hematopoiesis	NA	NA	NA
Location of human Thymopoiesis	NA	NA	NA
Reproducibility of engraftment (% mice engrafted)	NA	NA	NA
Identity of specific human leukocytes present	NA	NA	NA
Populated tissues	HIV provirus and infectious HIV produced by CD4 lymphocytes, macrophages, DC and microglia in all organs analyzed	Human transgenes expressed in rat CD4 lymphocytes, macrophages and microglia in all tissues analyzed	Mouse CD4 T cells and monocyte lineages, including macrophages, dendritic cells, and microglia
**Characteristics of HIV Infection of Humanized Rodent Models**			
HIV-specific immune response	None	Robust seroconversion, cellular responses not analyzed.	not examined
Tropism/clade of infecting HIV	R5- HIV-JR-CSF	R5 HIV-1 (YU-2 and V3 loop recombinant NL4-3) for CD4/CCR5-tg; NL4-3 for CD4/CXCR4-tg (unpublished)	R5 HIV strains (CCR5 Tg mice) and X4 strains (CXCR4 Tg mice)
Target cells infected	All cells	CD4 T-cells, macrophages	CD4+ T cells, macrophages, microglia
Level of plasma HIV viremia	10^2^~10^5 ^copies RNA/ml	2 × 10^2 ^RNA/ml (transient)	not observed
Duration of the infection	Life of the mouse	Low level viremia up to 7 weeks, low levels of 2-LTR circles at 6 months	not observed
Replication kinetics	Inducible by cellular activation	NA	NA
In vivo generation of ART resistance	NA	NA	NA
**Treatment of HIV Infection Using Humanized Rodent Models**			Not examined due to lack of replication in vivo
ART to block transmission	NA	Pre-EP and post-EP for efavirenz, enfuvirtide	NA
Microbicide to block transmission	NA	NA	NA
ART to control replication	NA	NA	NA
Emergence of resistance to ART	NA	NA	NA
Elimination of HIV reservoirs	NA	NA	NA
HSC gene therapy to protect progeny cells	NA	NA	NA
CD4 T cell gene therapy to protect cells	NA	NA	NA
**Immune-based Therapy of HIV Infection Using Humanized Rodent Models**			
Preventive HIV vaccines	NA	In progress (humoral immunity)	NA
Treatment HIV vaccines	NA	NA	NA
Adoptive Anti-HIV Ig therapy	NA	NA	NA
Adoptive Anti-HIV CTL therapy	NA	NA	NA
Immunoadjuvent therapy	NA	NA	NA
**Investigation of HIV Pathogenesis**			Not yet examined due to lack of replication
Contribution of HIV genes to pathogenesis	yes	NA	NA
HIV-mediated CD4-depletion-lymphoid	yes	NA	NA
HIV-mediated CD4-depletion-mucosal	yes	NA	NA
Effects of co-factors on replication	yes	CD4, CCR5, CXCR4, CyclinT1	CD4, CCR5, CXCR4, Cyclin T1, DC-SIGN
Effects of co-infection e.g. mTb on replication	yes	NA	NA
End organ dysfunction	yes	NA	NA

NA = not applicable

These transgenic mice were developed to also study the pathogenesis of HIV-1 infection. Early in the course of HIV infection, CCR5+CD4+ T cells are depleted from the lamina propria in HIV-infected individuals which is not reversed despite treatment with HAART. Critical questions that need to be addressed are the mechanism for this selective depletion of mucosal CD4 T cells, what interventions can reverse this depletion, and the role of dendritic cells and TH17 cells in this process. Development of a mouse model infectible with HIV would greatly support investigation of these critical questions. Future studies of the Littman group will focus on identifying barriers to gag processing in mouse cells, how to regenerate the CD4+ T cell population in the mucosal associated lymphoid tissues (MALT) after HIV infection and the role of dendritic cells in HIV infection of MALT.

Dr. Goldstein discussed an alternative approach used in his laboratory to construct mice that are transgenic for a provirus encoding a full length primary R5-tropic isolate, HIV-1_JR-CSF_, capable of producing HIV proteins and infectious virus, JR-CSF mice (Table 1) [18]. To circumvent the restricted trans-activating function of the Tat protein in mice and to specifically target HIV replication to CD4-expressing cells, Dr. Goldstein crossed the JR-CSF mice with transgenic mice that carry a transgene of hu-CycT1 under the control of the CD4 promoter and express hu-CycT1 in CD4 T cells, monocytes/macrophage dendritic cells and microglia to yield JR-CSF/hu-CycT1 mice [19]. As a consequence of being able to support Tat-mediated transactivation in CD4-expressing cells, HIV production is markedly increased in the JR-CSF/hu-CycT1 mouse CD4 T cells, monocytes and microglia. Stimulated JR-CSF/hu-CycT1 mouse CD4 T cells produced between 1- to 10% of the quantity of HIV produced by activated JR-CSF/hu-cycT1 mouse monocytes, indicating that mouse T cells have a specific block in post-HIV replication that is absent in mouse monocytes. While the population of peripheral CD4 T lymphocytes in the peripheral blood of JR-CSF mice remained stable over time, the peripheral CD4 T cells population in the JR-CSF/hu-cycT1 mice became gradually depleted so that by one year of age the CD4 to CD8 T cell ratio in the peripheral blood of the JR-CSF/hu-cycT1 mice had reversed to less than one, similar to the temporal course in HIV infected individuals that develop AIDS. In addition to being useful for studying the pathogenesis of HIV-mediated depletion of CD4 T lymphocytes in lymphoid tissues, these mice can also be used to investigate the in vivo effects of HIV infection on other organs, including the brain. The Goldstein group demonstrated that microglia and astrocytes from the JR-CSF/hu-cycT1 mice are more sensitive to in vivo activation by inflammatory stimuli such as LPS than are microglia from JR-CSF mice or wild-type littermates that is manifested by more extensive phenotypic changes and increased production of chemokines including of MCP-1. These mice provide a useful model for investigating the direct and indirect long-term effects of HIV-infection on cellular and organ function.

Dr. Keppler is pursuing the goal of humanizing rats to generate an immunocompetent multi-transgenic rat model of HIV-1 infection (Table 1). While cells from native rodents do not or only inefficiently support distinct steps of the HIV replication cycle, rats appear to be intrinsically more permissive than mice for supporting HIV replication. Of conceptual importance, the barriers to HIV replication in rat cells identified thus far appear to result from the inability of individual rat proteins to support HIV-1 replication rather than from the action of species-specific restriction factors. To circumvent these barriers, the Keppler group has pursued a block-by-block approach to humanize Sprague Dawley rats by the introduction of human transgenes that encode proteins that are required to overcome these barriers. Transgenic rats that express the HIV receptor complex hCD4 and hCCR5 on CD4 T-cells, macrophages and microglia (hCD4/hCCR5 rats) can be infected systemically with HIV [20,21]. Following intravenous challenge with HIV-1, lymphatic organs from hCD4/hCCR5 rats contained HIV cDNAs and early viral proteins, demonstrating successful *in vivo *infection. Furthermore, hCD4/hCCR5 rats infected with HIV_YU2 _displayed low-level plasma viremia (~150 copies/ml) for up to 7 weeks post-challenge as well as episomal HIV cDNA species in splenocytes and thymocytes 6 months post-infection. A recent proof-of-principle study showed the suitability of these double-transgenic animals for the rapid preclinical evaluation of the inhibitory potency and of pharmacokinetic properties of antiviral drugs targeting HIV entry or reverse transcription [22]. Prophylactic administration of Sustiva (efavirenz) or Fuzeon (enfuvirtide, T20) markedly inhibited the level of HIV infection measured several days after *in vivo *challenge with HIV. Additional novel drugs, including an integrase inhibitor, are currently being tested. In contrast, administration of a semen-derived fibril-forming peptide that has been shown by the Kirchhoff group to promote *in vitro *HIV infection increased the splenic HIV cDNA load in hCD4/hCCR5 rats after *in vivo *HIV challenge by 4.5 fold [23]. In their attempts to further enhance the HIV susceptibility of transgenic rats, the limited support of HIV replication at the transcriptional level that leads to reduced early HIV gene expression in rat T-cells was largely surmounted by the transgenic expression of a third human transgene, the Tat-interacting protein hCyclin T1, a component of the P-TEFb transcription complex [20]. T-cells from triple-transgenic rats produced 3-fold higher levels of HIV early gene products than rats transgenic only for hCD4 and hCCR5. However, robust replication is still precluded, most probably due to a disproportional representation of Rev-dependent HIV RNAs and viral proteins. The current work of the Keppler group focuses on the identification of a relevant factor that may overcome this third and possibly final barrier to HIV replication in primary target cells in rats. As a complementary approach, the Keppler group is pursuing strategies to adapt HIV to replicate in primary T-cells from transgenic rats.

## Chimeric human/mouse models

The generation of humanized mice for HIV research has benefited from a progression of genetic modifications made possible by the occurrence of spontaneous immunological mutations, the targeting of genes required for the development of innate and adaptive immunity, and the availability of inbred mouse strains exhibiting depressed innate immunity. The first widely used model for human hematolymphoid engraftment and subsequent HIV infection was the CB17-*Prkdc*^*scid *^(abbreviated as *scid) *mouse. CB17-*scid *mice supported engraftment with human (HSC), peripheral blood mononuclear cells (PBMC), and human fetal tissues. However, levels of engraftment were limited by many factors including host natural killer (NK) cell activity, spontaneous generation of mouse lymphocytes (leakiness), and the occurrence of spontaneous thymic lymphomas [24]. The subsequent development of the NOD-*scid *mouse stock exhibiting depressed NK cell activity resulted in heightened support of human hematolymphoid engraftment [24,25]. Humanized mice were incrementally improved over the next decade by the targeting of genes at a number of loci including the recombination activating genes 1 and 2 *(Rag1 *and *Rag2) *and the beta 2 microglobulin (*B2m*) locus [26]. Mutations at the *Rag-1 *and *Rag2 *loci prevent development of mature mouse lymphoid cells but do not reduce NK cell activity. The *B2m *mutation prevents NK cell development. Although NOD-*scid B2m*^*null *^mice lack NK cell activity, a shortened lifespan due to early occurrence of thymic lymphomas and other pathologic changes limited the use of this model in HIV research [27].

A major advance in development of humanized mice was made possible by the targeting of the gene encoding the interleukin-2 receptor common gamma chain (*Ilrg*), abbreviated as *IL2Rγ*. The IL2Rγ chain is indispensable for IL-2, IL-4, IL-7, IL-9, IL-15, and IL-21 high affinity binding and signaling [28]. The IL2Rγ mutation prevents NK cell development and causes other defects in innate immunity as well as depressed adaptive immunity. In humans, *IL2Rγ *deficiency causes X-linked SCID [29]. Four different groups independently targeted the mouse *IL2Rγ *gene [30-33]. Genetic crosses of *IL2Rγ *^*null *^mice with *scid, Rag1*^*null *^and *Rag2*^*null *^mice on a several different mouse strain backgrounds resulted in a number of new immunodeficient models that support engraftment with human HSC, PBMC, and fetal tissues [26].

### NOD/SCID/IL2Rγ^*null *^mouse model

Dr. Shultz and his colleagues, Drs. Dale Greiner and Fumihiko Ishikawa generated mice chimeric for the human hematopoietic system using one of the most widely available of the *IL2Rγ *deficient mouse stocks, the NOD-*scid Il2rg*^*tm*1*wjl *^(NOD-*scid IL2Rγ*^*null*^) model (Table 2). These mice lack mature lymphocytes and NK cells, survive beyond 16 months of age, and do not develop lymphomas [34]. The Shultz group demonstrated that newborn [12] and adult [34] NOD-*scid IL2Rγ*^*null *^mice support high levels of engraftment with human umbilical cord blood (UCB) HSC and mobilized HSC. The human HSC engrafted mice develop mature human lymphoid and myeloid cells and mount a humoral immune response to thymic-dependent antigens [11,12]. Engraftment of NOD-*scid IL2Rγ*^*null *^mice with either human committed lymphoid or myeloid progenitor cells isolated from human UCB results in development of both human conventional and plasmacytoid dendritic cells [35]. Adult NOD-*scid IL2Rγ*^*null *^mice also support heightened engraftment with human PBMC following intravenous, intraperitoneal, or intrasplenic injection [36]. Current ongoing genetic modifications of the NOD-*scid IL2Rγ*^*null *^model in Dr. Shultz's lab include further reductions of innate immunity as well as transgenic expression of human HLA molecules, cytokines, and other components needed to optimize human hematolympoid engraftment and function.

**Table 2:** SCID Mouse and NOD/SCID mouse-based chimeric human models

	**Dr. Stoddart**	**Dr. Shultz**	**Dr. Garcia-Martinez**
**Characteristics of Humanized Rodent Models**			
Strain	C.B-17 scid/scid (Taconic)	NOD-SCID IL2r gamma -/-	NOD/SCID
# mice/donor	50–60 mice/donor	CD34+ cell isolation yields 1 × 10^6 ^cells/donor sufficient for engrafting 20- to 25 mice	25
Source of human cells	Human fetal liver and thymus (20–24 g.w.)	Umbilical cord blood; mobilized hematopoeitic stem cells	Fetal liver/thymus
Method of isolation	not applicable	Magnetic bead enrichment	Magnetic beads
Pre-transplant treatment-mice	None	100 cGy for newborns; 325 cGy for adults; Intravenous injection	325 rads
Pre-transplant treatment-cells	None	None	None
Time frame from construction to experimental use	18 weeks	12 weeks	8–12 weeks
Location of human hematopoiesis	Thy/Liv organ	Bone marrow	Bone marrow
Location of human Thymopoiesis	Thy/Liv organ	Mouse thymus	Human thymic tissue
Reproducibility of engraftment (% mice engrafted)	90–100% with >80% CD4+CD8+	>90% of newborn and adult mice are engrafted in the bone marrow, spleen and thymus	>95%
Identity of specific human leukocytes present	Immature and mature T cells, B cells, macrophages, plasmacytoid DCs	B cells, T cells, conventional and plasmacytoid DCs, macrophages, monocytes, RBCs, platelets	T and B cells, DCs, monocytes/macrophages, NK, NKT and Tregs
Populated tissues	Human Thy/Liv organ	Bone marrow, thymus, spleen, lymph nodes, intestine, blood	GALT, Female and male reproductive tract, lung, bone marrow, lymph nodes, thymus, spleen, liver, peripheral blood.
**Characteristics of HIV Infection of Humanized Rodent Models**			
HIV-specific immune response	None reported	Work in progress	Yes, human IgG
Tropism/clade of infecting HIV	X4, R5, dual/mixed; clade B	Not tested	R5 and X4
Target cells infected	Intrathymic progenitors (CD3-CD4+CD8-), immature and mature thymocytes, macrophages	Not tested	CD4 T cells, monocytes/macrophages, DC
Level of plasma HIV viremia	None to highly variable	Not tested	Variable depending on stain of virus and tropism
Duration of the infection	5 weeks until severe depletion for X4 and dual/mixed; >6 months for R5	Not tested	Variable depending on stain of virus and tropism
Replication kinetics	Peaks at 3 weeks post infection (wpi) (X4 and dual/mixed), 6 wpi (R5)	Not tested	Isolate dependent
In vivo generation of ART resistance	Not observed for NL4-3 and 3TC (no RT M184V)	Not tested	Not done
**Treatment of HIV Infection Using Humanized Rodent Models**			
ART to block transmission	Not feasible	Yes	Not tested
Microbicide to block transmission	Not feasible	Yes	Not tested
ART to control replication	Yes, 4 classes of licensed ARVs so far.	Yes	Not tested
Emergence of resistance to ART	Not observed for NL4-3 and 3TC (no RT M184V)	Not done	Not tested
Elimination of HIV reservoirs	Not performed	Not done	Not tested
HSC gene therapy to protect progeny cells	Not performed	yes	Not tested
CD4 T cell gene therapy to protect cells	Not performed	Not done	Not tested
**Immune-based Therapy of HIV Infection Using Humanized Rodent Models**			
Preventive HIV vaccines	Not feasible	Yes	Not tested
Treatment HIV vaccines	Not feasible	Not done	Not tested
Adoptive Anti-HIV Ig therapy	Feasible, but not performed	Not done	Not tested
Adoptive Anti-HIV CTL therapy	Feasible, but not performed	Not done	Not tested
Immunoadjuvent therapy	Not feasible	Not done	Not tested
**Investigation of HIV Pathogenesis**			
Contribution of HIV genes to pathogenesis	Nef, Env (coreceptor usage), protease	Yes	Not tested
HIV-mediated CD4-depletion-lymphoid	Thy/Liv organ	Yes	Not tested
HIV-mediated CD4-depletion-mucosal	Not applicable	Yes	Not tested
Effects of co-factors on replication	Not determined	Yes	Not tested
Effects of co-infection e.g. mTb on replication	Not determined	Yes	Not tested
End organ dysfunction	Thy/Liv organ undergoes severe thymocyte depletion	Yes	Not tested

### RAG2^-/-^γ_c_^-/- ^mouse models

Dr. Akkina generated mouse-human chimeric mice using fresh CD34+ HSC isolated from human fetal liver cultured with cytokines for 1 day at a low density and injected hepatically (250,000 CD34+ cells/mouse) into RAG2^-/-γ^_c_^-/- ^Balb/c mice obtained from Dr. Irving Weissman (RAG-KO mice) (Table 3) [10]. Within the first 3 days of life, neonatal RAG-KO mice are sublethally irradiated and injected intrahepatically with CD34+ human hematopoietic stem cells to yield RAG-hu mice. After 8–12 weeks, the peripheral blood of the RAG-hu mice is populated with human T cells (CD4+, CD8+), B cells, dendritic cells, and macrophages. The fraction of CD45+ leukocytes detected in the peripheral blood of the RAG-hu mice of human origin was between is 5–80% and the Akkina group now routinely generate RAG-hu mice where the fraction of human peripheral blood lymphocytes is greater than 30% and human CD45+ leukocytes populate the mouse primary and secondary lymphoid organs. In addition, human T cells, macrophages and dendritic cells were detected in the vaginal, rectal and intestinal mucosa [37]. In the RAG-hu mice, human hematopoiesis continues for more than 1 year as evidenced by the maintenance of a stable population averaging 20–50% of human C45+ leukocytes in the peripheral blood.

**Table 3:** Rag2-/-γc-/- Mouse-based Human Chimeric Model

	**Dr. Akkina**	**Drs. Speck and Luban**	**Dr. Su**
**Characteristics of Humanized Rodent Models**			
Strain	Balb/c-Rag2-/-γ c-/-	Balb/c-Rag2-/-γ c-/-	Balb/c-Rag2-/-γ c-/-
# mice/donor	40/donor	CD34+ cell isolation yields 1–2 × 10^6 ^cells/donor sufficient for 5–10 mice (1 litter)	20–50/donor
Source of human cells	Fetal liver	Cord blood	Fetal liver
Method of isolation	Magnetic bead enrichment for CD34+ cells	Magnetic bead enrichment for CD34+ cells	CD34+ MACS kit
Pre-transplant treatment-mice	Irradiation 350 rads; intrahepatic injection into newborns	Irradiation 200 rads given twice 4 h apart; intrahepatic injection into newborns	Irradiation 400 rad; intrahepatic injection into newborns
Pre-transplant treatment-cells	SCF, IL-3, IL-6	None	None or retroviral transduction
Time frame from construction to experimental use	12 weeks	12–16 weeks	>12 weeks
Location of human hematopoiesis	Bone marrow	Not investigated	BM, Spleen, LN
Location of human Thymopoiesis	Mouse thymus	Not investigated	Mouse thymus
Reproducibility of engraftment (% mice engrafted)	>95%	More than 90% of mice show human cells in periphery; about 50% of mice have levels >10% huCD45+ cells	>95% with >20% human CD45+ cells in blood
Identity of specific human leukocytes present	T and B cells, DCs, monocytes/macrophages and some granulocytes	B and T cells, monocytes, DCs	All human leukocytes
Populated tissues	Bone marrow, lymph nodes, thymus, spleen, liver, intestines, lungs	Thymus, spleen, blood, MLN, BM, liver; to some extent: gut	BM/thymus/spleen/LN (no significant Peyer's patches found)
**Characteristics of HIV Infection of Humanized Rodent Models**			
HIV-specific immune response	Not detected	Some minor B cell response (1/25 animals tested); no T cell response detected	Low gag-specific responses/no IgG detected
Tropism/clade of infecting HIV	R5, X4, dual-tropic	YU-2 and NL4-3	R5-X4-dual or R5/clade B
Target cells infected	CD4 T cells	CD3+ cells and only occasionally non T cells such as CD68+ macrophages	CD4 T and DC
Level of plasma HIV viremia	~10^7 ^copies RNA/ml	Up to 2 × 10^6 ^copies/ml	10^5^-10^6 ^copies/ml
Duration of the infection	at least 14 months	Up to 190 days; longest period followed	>22 weeks
Replication kinetics	Peak viremia at about 6 weeks followed by maintenance of viremia	HIV RNA levels peak 2–6 wpi, thereafter viremia mostly stabilizes at lower levels.	HIV RNA levels peaks at 2–3 (dual tropic) or 4–6 wpi (R5-tropic)
In vivo generation of ART resistance	Not done	Not tested	Not known
**Treatment of HIV Infection Using Humanized Rodent Models**			
ART to block transmission	Not done	Not done	Not done
Microbicide to block transmission	Not done	Not done	Not done
ART to control replication	Not done	Not done	Yes.
Emergence of resistance to ART	Not done	Not done	Not done
Elimination of HIV reservoirs	Not done	Not done	Not done
HSC gene therapy to protect progeny cells	yes	Not done	Not done
CD4 T cell gene therapy to protect cells	Not done	Not done	Not done
**Immune-based Therapy of HIV Infection Using Humanized Rodent Models**		Not done	
Preventive HIV vaccines	Not done	Not done	Not done
Treatment HIV vaccines	Not done	Not done	Not done
Adoptive Anti-HIV Ig therapy	Not done	Not done	Not done
Adoptive Anti-HIV CTL therapy	Not done	Not done	Not done
Immunoadjuvent therapy	Not done	Not done	Not done
**Investigation of HIV Pathogenesis**			
Contribution of HIV genes to pathogenesis	yes	Not done	Env/Nef
HIV-mediated CD4-depletion-lymphoid	yes	yes	Yes
HIV-mediated CD4-depletion-mucosal	Not done	Not done	mesenteric LN, yes
Effects of co-factors on replication	Not done	Not done	Not done
Effects of co-infection e.g. mTb on replication	Not done	Not done	Not done
End organ dysfunction	Not done	Not done	Not done

The RAG-hu mice are infectible with a variety of X4 and R5 isolates, have plasma viremia and circulating human PBMC containing HIV that is detectible by PCR [10]. The level of viremia in the plasma ranged up to 165,000–12,200,000 copies/ml, but may rise and fall over time. HIV infection is detected in lymphoid tissues and CD4 depletion occurs after HIV infection, but the extent of CD4 depletion can vary widely during infection. The infection has persisted for a long time, with HIV detected in the RAG-hu bone marrow almost 1 year after inoculation. Mucosal HIV transmission occurred in RAG-hu mice as evidenced by the development of plasma viremia within 1 week after the mice were infected vaginally and rectally with an R5 HIV isolate without any prior hormone treatment or introduction of mucosal abrasion. This primary transmission was associated with the dissemination of infection to mouse lymph nodes, intestines and spleen. X4 HIV isolate was also found to be capable of mucosal transmission via both vaginal and rectal routes although the efficiency of infection was lower than R5 virus. Mucosally infected RAG-hu mice displayed CD4 T cell depletion, but depletion occurred later and was not as dramatic as seen in mice after intraperitoneal infection. Advantages of the RAG-hu model for studying HIV infection include its capacity to support chronic productive HIV infection for over 1 year, to display CD4 depletion and to being susceptible to infection by either vaginal or rectal routes.

HIV infection may undermine the human immune response of RAG-hu mice. Studies using the RAG-hu mouse system by the Akkina group to model Dengue fever, for which there is currently no ideal animal model available to study viral pathogenesis and to test vaccines, may be more informative of the capacity of RAG-hu mice to generate primary human immune responses [38]. There are 4 serotypes of Dengue virus and re-infection of individuals with a second serotype virus causes worse disease than infection with the primary virus due to antibody-dependent enhancement. After challenges of RAG-hu mice with Dengue virus the mice become infected and develop Dengue-specific antibody. Viremia (10^6 ^particles/mL) lasts up to 2 weeks and Dengue viral replication is detected in the mouse spleens. Dengue-specific IgM and IgG responses are first detected at 2 weeks and at 6 weeks after infection, respectively. Dengue virus neutralization was detected in the sera of some mice at a titer of up to 1,000 by using a FACS-based assay. Of interest was the observation that the immune response to Dengue was much more robust than the immune response to HIV after infection. This may reflect HIV-associated compromise of the human immune system in the RAG-hu mice.

Future studies by the Akkina group using this model will include evaluating the long-term effects of microbicides, studying viruses that infect the hematolymphoid system, evaluating gene therapy strategies using vectors carrying anti-HIV genes and drug-selection makers, investigation of the mechanism of antibody-dependent enhancement during Dengue infection and the testing of Dengue vaccines.

Dr. Luban reported on the system developed by Markus Manz at his Institute, of injecting human CD34+ HSC intrahepatically into newborn Balb/c RAG2^-/-γ^c^-/- ^mice (Table 3) [15]. These mice were obtained from Dr. Weissman, who originally got them form Dr. Mamoru Ito in Japan. Strain-specific factors contributed to the degree of reconstitution. Mice carrying the same RAG2 and γ c deletions on the C57BL/6 background did not become reconstituted with human leukocytes. In contrast, the lymphoid tissues of the Balb/c RAG2^-/-γ^c^-/- ^mice display reconstitution with human B cells and T cells and population of the thymus with human T cells. No significant population of human leukocytes was detected in the mouse mucosa or brain. After intra-peritoneal injection of either the R5 or X4 strains of HIV, YU2 and NL4-3, respectively, the mice developed systemic infection with sustained plasma viremia of up to 10^6 ^HIV RNA copies/ml [39].

The γ_c_^-/- ^mice used in these studies have a partial deletion of the common gamma chain receptor gene with expression of a truncated common gamma chain receptor that binds the appropriate cytokine, but lacks the intracellular signaling region. It is unclear if this truncated receptor has any functional activity, but mice having complete deletion of the common gamma chain receptor are also available. The litter size of the Balb/c RAG2^-/-γ^c^-/- ^mice ranges from 3 to 11 mice, with an average of about 6 mice. Their group obtains sufficient human CD34+ HSC from each cord blood donor to inject an average of 4–6 mice. After reconstitution of the mice, analysis of whole blood after RBC lysis, demonstrated that the peripheral blood of 90% of the mice was populated with >5–10% human CD45+ cells. An aliquot of cord blood yields an average of 5 × 10^5 ^CD34+ cells, with a range of 2 × 10^5 ^– 2 × 10^6 ^cells. Cord blood from separate donors can be pooled and one donor provides sufficient human CD34+ cells to reconstitute one litter of mice. The CD34+ cells can be frozen and, in fact, the majority of their mice are reconstituted with frozen cells.

Advantages of this model are that it uses no human fetal tissues, requires no surgery, and displays no global activation of the human leukocytes populating the mouse lymphoid tissue. The mice are hardy, breed well and develop no tumors in the thymus. These mice can be used to evaluate therapeutics, but their use for this purpose is limited by the modest throughput. They can be used to study HIV pathogenesis including which isolates infect brain and other cell types, mechanisms of cell to cell spread, restriction factor biology, and potentially in vivo imaging. They also can be used to study the impact of viral genetic and host genetic factors on HIV replication. This relatively facile reconsitution model may thus be an ideal system in which to test antiviral gene therapy.

The human T cells are likely to mature in the mouse thymus and interact with human dendritic cells that are present in the mouse thymic epithelial tissues. Positive selection is indicated by the presence of mature human T cells in the periphery and negative selection is indicated by the absence of graft vs. host disease [40].

After infection with HIV, low liters of HIV-specific antibody are detected in only 1 in 25 mice [39]. To use these mice as a model to study vaccines needs improvement. The low number of CD34+ HSC that can be isolated from the cord blood limits the number of mice that could be generated from isogenic CD34+ cells. Over several months, the levels of human CD45+ cell declined, and human CD45+ leukocytes were not detected in the mucosa or lungs of the mice. The capacity of human leukocytes to mature, differentiate and localize to the appropriate lymphoid tissue may be limited by the inability of some mouse molecules to exert their functional activity on human cells. The current mouse model permits introduction of gene therapy vectors into human HSC, prior to injection into the mice, including genes that could protect mature human CD4 T cells from HIV infection. For this purpose the Luban group is developing lentiviral vectors. To circumvent the limited availability human HSC derived from cord blood, Dr. Luban is attempting to generate human HSC from human ES cells.

Dr. Su uses the same Balb/c RAG2^-/-γ^c^-/- ^mouse (DKO mouse) but circumvents the limitation of the low number of CD34+ HSC obtainable from cord blood by using human fetal liver as a source of human HSC (Table 3). After intrahepatic injection of neonatal DKO mice with human CD34+ cells (5 × 10^5 ^cells/mouse), the periphery of the mice (hu-DKO mice) become populated with human T cells, B cells and dendritic cells, including mDc and pDC. The human leukocytes populate the mouse spleen to about 1/3 the size of the normal mouse spleen and the mouse thymus to about 20% of the size of the normal mouse thymus. The human T cells undergo positive and negative selection during maturation as indicated by the observation that the human cell-tropic EBV infection leads to effective anti-EBV T cell responses and circulating human T cells (or splenocytes) do not generate a mixed lymphocyte reaction (MLR) against human leukocytes from another hu-DKO mouse transplanted with human CD34+ HSC from the same donor but do generate an MLR against human leukocytes isolated from a hu-DKO mouse transplanted with CD34+ HSC from a different donor. Although mesenteric nodes draining the intestines of the hu-DKO mice contained human T cells and B cells, they did not detect either Peyer's patches or significant numbers of human CD45+ leukocytes in the lamina propria of the gut. Immunization of the mice with an HBV vaccine generated germinal centers in the mouse lymph nodes. Although the mouse B cells do not express IgG, they can be driven in vitro to produce IgG if incubated with T cells stimulated with anti-CD3 and anti-CD28. The population of human T cells is not increased phenotypically in number by implantation of human thymic tissues from the same donor, but the implanted mice subsequently developed rashes and became sick.

The hu-DKO mice were challenged by the Su group with HIV through several routes. After intravenous infection with the HIV isolate R3A, 100% of the mice become infected. If the mice are challenged with cell-free HIV by intrarectal inoculation, none of the mice became infected. Intrarectal challenge with HIV-infected cells caused transient infection that was cleared by 3 weeks after inoculation. When intrarectal inoculation with HIV-infected cells was accompanied with mucosal injury, sustained HIV infection occurred that was associated with depletion of the peripheral human CD4 T cells. During the course of infection, the level of plasma virus fluctuated and correlated inversely with the number of human CD4 T cells in the peripheral blood. The hu-DKO mice were used to examine the role of HIV-induced immune activation in mediating CD4 depletion. The Su group examined the role of CD4 Treg cells in HIV infection using the hu-DKO mice. HIV infects and replicates efficiently in CD4 Treg cells. In the hu-DKO mice, about 3–5% of circulating CD4 T cells were CD25+ FoxP3+, the phenotype of CD4 Treg cells. These CD4 Treg cells are functional, as evidenced by their capacity to suppress T cell proliferation in an in vitro assay. In hu-DKO mice, the R3A isolate of HIV infects and rapidly depletes CD4 Treg cells. HIV-mediated depletion of Treg with loss of their inhibitory regulatory activity may contribute to HIV-mediated hyperimmune activation.

Dr. Speck presented his group's experience using hu-DKO mice generated from Balb/c RAG2^-/-γ^c^-/- ^mice (DKO mice) injected with human CD34+ HSC isolated from cord blood either fresh or frozen and stored at -80°C (Table 3). Close to 100% of the lymphocytes present in the lymph node and thymus of the time were human. There was also a large quantity of human dendritic cells in the liver. After inoculation with HIV strains YU2 or NL4-3, the mice developed sustained high levels of viremia, and displayed CD4 depletion that was more extensive after infection with NL4-3 than with YU2 [39]. In the thymus, extensive infection with NL4-3 was detected, but almost no infection with YU2 was observed. About 1 in 25 mice generated HIV-specific antibodies. While construction of the hu-DKO mice did not require the use of fetal tissue and the hu-DKO mice developed sustained and disseminated HIV infection, the infected hu-DKO mice generated almost no HIV-specific immune responses. The mice displayed variable rates of engraftment and the function of the engrafted leukocytes may vary depending on unique genetic factors associated with the specific donor of the CD34+ HSC. Use of these mice requires a BSL2/3 facility and access to cord blood. Dr. Speck's group can generate about 50 mice/month. Engraftment depends on the quality of the mice and for optimal engraftment new breeding pairs are used every 3–4 months. Engraftment with human leukocytes decreases over time and the percentage of circulating human T cells needs to be above 1% to get successful infection after intraperitoneal injection. However, the level of engraftment with human CD45+ leukocytes does not predict the level of HIV replication after infection.

Despite the generation of Ag-specific immune response to model antigens, the hu-DKO mice displayed no or very weak adaptive immune responses to HIV. They also did not display hypergammaglobinemia, a hallmark of early HIV infection. Only scattered human CD45+ leukocytes were detected in the gut mucosa. After treatment of the mice with progesterone, the mice exhibited thinning of the vaginal epithelia and could be infected by the vaginal route with HIV-infected PBMC but not with cell-free virus. No human T cells were detected in the lamina propria and human CD45+ leukocytes detected in the perianal tissues were likely to be human macrophages.

### NOD/SCID-based mouse models

Dr. Victor Garcia presented his results using a different model, BLT mice (Table 2) [17,41] where human fetal liver and thymus are divided into pieces that are surgically implanted under the kidney capsule of NOD/SCID mice; one fraction of the liver is used for isolation of CD34+ HSC that are frozen and three weeks after surgery, the mice are sublethally irritated and intravenously injected with the freshly thawed syngeneic CD34 HSC (0.25–2.5 × 10^6 ^cells/mouse). One-to-two months after injection, 20–50% of the circulating leukocytes in the peripheral blood of the mice are human, and include human T cells, B cells, monocytes and dendritic cells. The human T cells in the peripheral blood express a broad range of Vβ TCRs. Functional activity of the human DC was indicated by the selective expansion of the human Vβ 2+ T cell population after administration of TSST-1 with subsequent production of TNF-γ, IFN-γ, IL-10, IL-6, IL-2, and IL-8 after 18 hours. After infection with EBV, the mice show an expansion of the CD45RA-CD27+ memory T cells and the emergence of human T cells that could generate IFN-γ ELISPOTs in an MHC-restricted manner to autologous EBV-infected lymphoblastoid cells. Functional activity of the human lymphocytes was indicated by the observation that in contrast to EBV-infected NOD/SCID mice, EBV-infected BLT mice did not develop EBV-induced tumors. The intestine and rectum of the BLT mice were populated with human CD4+ T cells. No human cells were detected in the brains of the BLT mice. The small intestines of the mice were populated with human intraepithelial lymphocytes (IEL) expressing CD8 α and β chains and lamina propria lymphocytes (LPL) that only expressed the CD8 alpha chain. The number of human CD4+ T cells exceeded the human CD8+ T cells in the small intestine but not in the large intestine. The human CD4+ T cells expressed low levels of CXCR4 and higher levels of CCR5. After mice were challenged with the LAI strain of HIV, they became infected as indicated by the presence of p24 antigen in the plasma. Three of four infected mice had human antibodies to gp120, p66 and p24 detected by Western blot. The target cells for HIV infection were CD3+ CD4+ T cells and after HIV infection there was almost complete depletion of CD4+ T cells in the mesenteric lymph node and CD4+ and CD8+ T cells in the small intestine lamina propria. Intrarectal infection with the JR-CSF stain resulted in systemic HIV infection of the mice, but the CD4+ T cell depletion was not as dramatic as that observed after intravenous infection. Systemic HIV infection could also be introduced by intravaginal installation of both X4 and R5 isolates of HIV. This permitted the Garcia group to use these mice to test the anti-HIV activity of microbicides. They demonstrated that pre-exposure prophylaxis with a proprietary microbicide completely protected the mice (n = 5 mice) from systemic HIV infection after intravaginal inoculation with HIV-1.

### SCID Mouse-based mouse models

Dr. Stoddart presented her experience using the SCID-hu Thy/Liv- mouse model (Table 2) [42]. This model, first described by Dr. McCune, consists of SCID mice implanted with syngeneic pieces of human fetal liver tissues by surgical placement under their kidney capsules [5]. A single donor provides sufficient tissue to implant 50–60 mice. The mice are implanted at about 8 weeks of age, and by 18 weeks the implant grows into a tissue that resembles human fetal thymus with sufficient volume to infect by direct injection with HIV-1. This approach permits the generation of about 1,200 mice/year. After direct injection of HIV-1 into the implant, the thymic tissue becomes infected with HIV, but the infection does not disseminate outside the implant. After injection of the thymic implant with the HIV-1 X4-tropic strain NL4-3, the mice do not develop reproducible plasma viremia, and implant viral loads peaks about 3 weeks after inoculation. Dr. Stoddart demonstrated that the dose response and antiviral activities of licensed ART in the SCID-hu model was comparable to that observed during treatment of HIV-infected individuals. A major advantage of the SCID-hu model is that large cohorts of mice can be constructed from the same donor removing a potential confounding variable in comparative drug studies of the differential response of tissues from different donors. This also permits the production of mice at a low cost per mouse. The SCID-hu mice can be infected with wild type or mutant isolates of HIV-1 for the evaluation of anti-HIV therapy against wild-type or drug-resistant mutants or investigation of the impact of mutations in HIV genes on in vivo replicative capacity. Use of the SCID-hu Thy/Liv model is limited by the requirement for fetal tissue, the need for surgical construction of each mouse, the lack of immune responses generated by the human T cells, and the limitation of HIV infection to the human thymic implant.

## Perspective and Recommendations

Dr. KewalRamani summarized the advantages and disadvantages of studying HIV infection using rodent-based models compared to macaque-based models. Advantages of the human chimeric mouse models are that they are populated with human lymphoid and myeloid cells, infectible with a broad range of HIV isolates, amenable to genetic manipulation of the mouse recipient, able to recapitulate variation among donor recipients and are relatively inexpensive. Disadvantages of the human chimeric models are their inability to generate a robust HIV-specific immune response which precludes their use for testing HIV vaccines, their requirement for human HSC and the lack of regional centers. Dr. Lifson discussed the selective infection of macaques with SIV and not HIV. Although the pathogenic behavior of various strains of SIV isolates closely resembles that of HIV including the induction of CD4 depletion, development of CNS disease and persistence of HIV in anatomical reservoirs during ART, experimental results using SIV-infected macaques may not correlate with the pathogenesis of HIV infection in humans. While experiments using SIV-infected macaques are expensive, the presence of regional primate centers provides investigators with access to this primate model.

A panel of investigators discussed the following Questions during Panel discussion:

What are the hindrances to widespread use of the models for pathogenesis studies?

What is needed to determine the feasibility of the models for vaccine and therapeutic testing, including testing of microbicides?

*What can be done to overcome some of the current blocks for the models to support quality assays? Some of these include CD34 cell suppliers, mice, how cells are treated, virus, others*.

What can the NIH do to facilitate solutions to the above questions?

A limiting factor discussed by panel members was the variable availability of these newer immunodeficient mice to investigators. This could be circumvented by having NIAID set up a centralized repository in an existing animal facility to permit establishing and replenishing mouse colonies for investigators. Another bottleneck is the availability of human HSC. Three sources of human HSC are available. The first is mobilized CD34+ HSC obtained from GM-CSF-stimulated individuals, a modality used to obtain HSC autologous bone marrow for transplant, particularly in individuals with leukemia. However, Dr. Su stated that in his lab, mobilized human HSC did not effectively engraft the DKO mice with human hematopoietic cells. The second source of human HSC is cord blood. While the number of HSC obtained from a cord blood donor is only sufficient for reconstituting 4- to 6 mice, cord blood is readily available and an NIH-sponsored repository could be set up that provide cord blood HSC to investigators. Furthermore, pooled CD34+ HSC from several cord blood donors could be used for some experiments requiring larger numbers of mice. The third human HSC source is human fetal liver, which provides sufficient numbers of CD34+ HSC to populate large quantities of mice. However, because the source of this tissue is from fetal abortuses, access to this tissue is restricted and subject to additional review by oversight committees.

The panel highlighted several areas that should be the subject of future scientific investigation to advance these model systems, particularly identification of factors that would increase the development of functional human immune response in these mice. Although population of the hu-DKO mouse thymus with human double and single positive T cells was suggestive of it being the location of human thymopoiesis, double positive T cells were also detected in lymph nodes. Therefore, further dissection of the mechanism of human T cell maturation in this system could provide new methods to increase the qualitative function of T cells that mature in the human mouse chimeric model. A critical technical issue was raised regarding the importance of standardizing metrics for measuring the population of human leukocytes in the peripheral blood to permit the repopulation results of different groups to be compared. To compensate for variation in gating for live cells or lymphocytes, one approach would be to report the ungated percentage of human CD45 cells after lysis of whole blood while another approach would be to report the population as the absolute number of human leukocytes/ml of blood. Another area to investigate is the mechanism of localization of mouse lymphoid and myeloid cells to the mouse mucosal tissue. While Dr. Akkina reported that the gut mucosal tissue of hu-DKO mice constructed by his group were populated with human leukocytes, relatively more extensive human leukocyte engraftment in the mucosal tissue has been documented for the humanized BLT mouse model [17,41]. Delineation of the basis for the differential capacity of human leukocytes to populate the mucosal associated lymphoid tissues of mice may provide insights into the mechanism for lymphocyte homing to the gut and other mucosal areas. For example, is it a consequence of the different location of maturation of the human leukocytes in the mouse models, human thymic implant vs. mouse thymus or due to the different immunodeficient mice used, NOD/SCID vs Rag^-/-^γ_c_^-/-^? The consensus of the investigators was that there is no clear-cut best rodent model that is applicable to all studies and specific models may be more suited for investigating different aspects of HIV pathogenesis and therapeutic efficacy. For this purpose it is advisable to continue to fund the development of new rodent model systems.
